# A Systematic Review of Predictions of Survival in Palliative Care: How Accurate Are Clinicians and Who Are the Experts?

**DOI:** 10.1371/journal.pone.0161407

**Published:** 2016-08-25

**Authors:** Nicola White, Fiona Reid, Adam Harris, Priscilla Harries, Patrick Stone

**Affiliations:** 1 Marie Curie Palliative Care Research Department, Division of Psychiatry, University College London, London, United Kingdom; 2 Department of Primary Care & Public Health Sciences, King’s College London, London, United Kingdom; 3 Department of Experimental Psychology, University College London, London, United Kingdom; 4 Department of Clinical Sciences, Brunel University London, London, United Kingdom; University of Exeter, UNITED KINGDOM

## Abstract

**Background:**

Prognostic accuracy in palliative care is valued by patients, carers, and healthcare professionals. Previous reviews suggest clinicians are inaccurate at survival estimates, but have only reported the accuracy of estimates on patients with a cancer diagnosis.

**Objectives:**

To examine the accuracy of clinicians’ estimates of survival and to determine if any clinical profession is better at doing so than another.

**Data Sources:**

MEDLINE, Embase, CINAHL, and the Cochrane Database of Systematic Reviews and Trials. All databases were searched from the start of the database up to June 2015. Reference lists of eligible articles were also checked.

**Eligibility Criteria:**

Inclusion criteria: patients over 18, palliative population and setting, quantifiable estimate based on real patients, full publication written in English. Exclusion criteria: if the estimate was following an intervention, such as surgery, or the patient was artificially ventilated or in intensive care.

**Study Appraisal and Synthesis Methods:**

A quality assessment was completed with the QUIPS tool. Data on the reported accuracy of estimates and information about the clinicians were extracted. Studies were grouped by type of estimate: categorical (the clinician had a predetermined list of outcomes to choose from), continuous (open-ended estimate), or probabilistic (likelihood of surviving a particular time frame).

**Results:**

4,642 records were identified; 42 studies fully met the review criteria. Wide variation was shown with categorical estimates (range 23% to 78%) and continuous estimates ranged between an underestimate of 86 days to an overestimate of 93 days. The four papers which used probabilistic estimates tended to show greater accuracy (c-statistics of 0.74–0.78). Information available about the clinicians providing the estimates was limited. Overall, there was no clear “expert” subgroup of clinicians identified.

**Limitations:**

High heterogeneity limited the analyses possible and prevented an overall accuracy being reported. Data were extracted using a standardised tool, by one reviewer, which could have introduced bias. Devising search terms for prognostic studies is challenging. Every attempt was made to devise search terms that were sufficiently sensitive to detect all prognostic studies; however, it remains possible that some studies were not identified.

**Conclusion:**

Studies of prognostic accuracy in palliative care are heterogeneous, but the evidence suggests that clinicians’ predictions are frequently inaccurate. No sub-group of clinicians was consistently shown to be more accurate than any other.

**Implications of Key Findings:**

Further research is needed to understand how clinical predictions are formulated and how their accuracy can be improved.

## Introduction

Studies show that patients, carers, and clinicians all value accurate prognostic information [1–6]. Prognostic accuracy is important at all stages of the illness trajectory [7]. When a prognosis is discussed openly, it can give family members, patients, and clinicians the opportunity to engage fully with each other, make informed decisions and receive specialist physical and emotional support in a timely manner [7, 8], particularly when the prognosis is short.

In the United Kingdom, a recent review of a care pathway for a dying patient called the Liverpool Care Pathway (LCP) [9], highlighted that clinicians are not very accurate at recognising which patients are imminently dying. This is in contrast to previous research which has suggested an “horizon effect” in prognostication [10]. The so-called “horizon effect” suggests that clinicians should be more accurate at recognising a shorter rather than a longer prognosis.

There have been three reviews published that have reported on the accuracy of clinician estimates which suggest that clinicians’ predictions about length of survival are inaccurate and unreliable [10–12]. These reviews have all been limited to patients with advanced cancer. Evidence for patients with a non-cancer diagnosis suggests that clinicians’ determinations of prognosis in these patients may be more inaccurate than those in cancer patients [13].

The most common method of predicting survival in clinical practice remains simple clinical intuition. In order to improve general clinicians’ prognostic skills it is important to learn from clinicians who have a particular expertise in this area. Which leads to the questions, are some clinicians better at prognosticating than others? Are there individual factors, such as professional training or years of experience that make a clinician a more expert prognosticator?

This review extends current literature by including all diagnoses and including all healthcare professionals. Using this approach, our final conclusion should be applicable to all disciplines who are asked to provide a prognosis.

## Aims

The systematic review questions were:

- How accurate are clinicians’ predictions of survival in palliative care patients?
- Are any subsets of clinicians more “expert” at prognostication than others?

## Methods

The protocol for this systematic review is available as supplementary material (S1 Appendix)

### Search Strategy

The search strategy was developed in line with the recommendations of the Cochrane Prognosis Methods Group [14]. The search strategies from previous literature [11, 15] were also referred to for guidance. Combined terms used were for: “Palliative care patients”; “Clinicians’ predictions”; and “Prognosis” (S2 Appendix). Sensitivity of the search strategy was tested by running the search and checking that key papers known to the authors were identified.

The databases searched were MEDLINE, Embase, CINAHL, and the Cochrane Database of Systematic Reviews and Trials. Searches were conducted from inception up to June 2015. A search of the reference lists of the final studies was also conducted.

Authors identified in the review were contacted and asked if they were aware of any unpublished literature in the area. A grey literature website [16] was searched for unpublished work.

### Inclusion/exclusion criteria

#### Inclusion

Studies were included in this review if all the following criteria were satisfied:

- Patients were over 18
- Patients were defined within the study as being “not curative”, “palliative”, or having a “terminal illness”
- The clinician making the prognostic estimate worked in a palliative care setting (i.e. a hospital or community palliative care team, or a hospice). A clinician, in this review, was defined as healthcare professional, such as a doctor (of any profession), a nurse, or any clinician who provides therapeutic support to a patient.
- Any study design in which a prognosis from a clinician was quantified either in terms of duration or probability of survival
- Written in English

#### Exclusion

Studies were excluded if any of the following criteria were satisfied:

- Animal study
- Age of the patients was less than 18 years
- The clinical setting was Intensive Care Unit (or similar) or patients were receiving artificial ventilation
- The study concerned assessment of prognosis following a specific intervention e.g. survival following surgery or chemotherapy
- Only published in abstract form
- The prognostic estimates were based on hypothetical cases rather than real patients.
- The prognostic questions were not quantifiable (e.g. I would not be surprised if this patient died within one year).

### Quality Assessment

Identifying prognostic studies and evaluating their risk of bias is challenging [15, 17]. We used the QUIPS tool to assess bias [18]. The domain of “Study Participation” was scored twice, in order to reflect the involvement of both clinician and patient populations within the same study. The tool was completed by one researcher (NW). In the event of any doubt about the score, an independent second reviewer (PS) discussed the study with the researcher.

It was decided that no study would be excluded based on the quality assessment score, in order to provide a full account of clinician survival estimates. For several of the studies identified, the accuracy of the clinical estimate of prognosis was not the primary outcome of the research, but was part of a secondary analysis. The QUIPS score of each paper has been reported for transparency but has not been used as a basis for exclusion.

### Data Extraction

Using a standardised table, one reviewer (NW) extracted information from each study regarding the setting, characteristics of clinicians, type of prognostic estimate (see below), and patient population. In the event of uncertainty, a second reviewer (PS) was consulted. In order to facilitate synthesis of data, studies were grouped according to the type of prognostic estimate obtained; categorical, continuous, or probabilistic (see below for definitions).

#### Categorical prognostic estimates

Categorical prognostic estimates occurred when clinicians were asked to pick from a pre-determined list of survival durations, e.g. 0–14 days, 15–56 days and >56 days, or the analysis had been reported using such categories. The raw data from each study were extracted; where percentage accuracy was given, the absolute number was calculated. The number of accurate estimates relative to the total number of estimates provided in the study was calculated. Accuracy, in this context, equates to the frequency with which the clinician selected the correct survival category.

#### Continuous prognostic estimates

Continuous prognostic estimates occurred when clinicians were asked an open question about how long a patient was expected to survive (e.g. how many days do you expect this patient to live?). The data from these studies were often reported as the median predicted and median actual survival. The outcome was usually reported in days, however in several papers, weeks were recorded. In order to keep the outcome the same across the studies, all estimates were converted to days. Accuracy, in this context, is defined as the difference between median predicted and median actual survival.

#### Probabilistic prognostic estimates

Probabilistic prognostic estimates occurred when clinicians were asked to determine the percentage likelihood of an outcome at a specified time-point (e.g. what is the probability that this patient will be alive in three months’ time?).

#### Relative prognostic accuracy of different types of clinicians

Information about the clinicians being evaluated (e.g. professional background, speciality training, and years of experience) and the types of prognostic estimate they were asked to undertake (categorical, continuous or probabilistic) were extracted where possible. Further categorisation by years of certification or speciality was not possible due to a lack of available information.

#### Missing data

When data were not presented fully in published reports, the study authors were contacted for more information [19–37]; three authors returned additional data [35–37]. For numerous studies providing continuous estimates, predicted and actual survival data were missing, and were no longer available from the study authors [22, 28, 29, 38, 39]. In these cases, we used summary results presented in a previous systematic review [11] which had managed to obtain the raw data before it had been destroyed. For those studies where the relevant data could not be obtained, the results were presented narratively.

### Data analysis

For studies with categorical prognostic estimates, a forest plot was created showing the accuracy of estimates as a percentage of the total number of estimates for each study. For studies with continuous prognostic estimates, a Professional Error Score (PES) was calculated for each study. The PES is the difference between the median predicted survival (PS) and the median actual survival (AS) as a percentage of the actual survival; (PS–AS)/AS)*100 [27], where 0 represents perfect accuracy. For studies with a probabilistic estimate, the data were described narratively.

Several papers presented accuracy in terms of the area under the Receiver Operating Characteristic (ROC), known as the c-statistic or the ‘ROC value’. These analyses are frequently used when assessing the accuracy of a diagnostic test. True positive rates (sensitivity) are plotted against false positive rates (1—specificity) to investigate whether clinicians can discriminate accurately between those who will and won’t die at particular time points. The closer the ROC value or c-statistic is to 1, the more accurate are the clinicians. As a general rule, a value of 0.5 suggests no ability to discriminate, a value of ≥0.7 and <0.8 an acceptable level of discrimination, ≥0.8 and <0.9 an excellent level of discrimination and ≥0.9 is outstanding.

Due to the degree of clinical heterogeneity between studies, it was deemed inadvisable to conduct a meta-analysis to calculate a pooled “overall” estimate, for any of the types of estimate considered (categorical, continuous or probabilistic).

STATA v13 was used for the data analyses.

## Results

**Fig 1:**
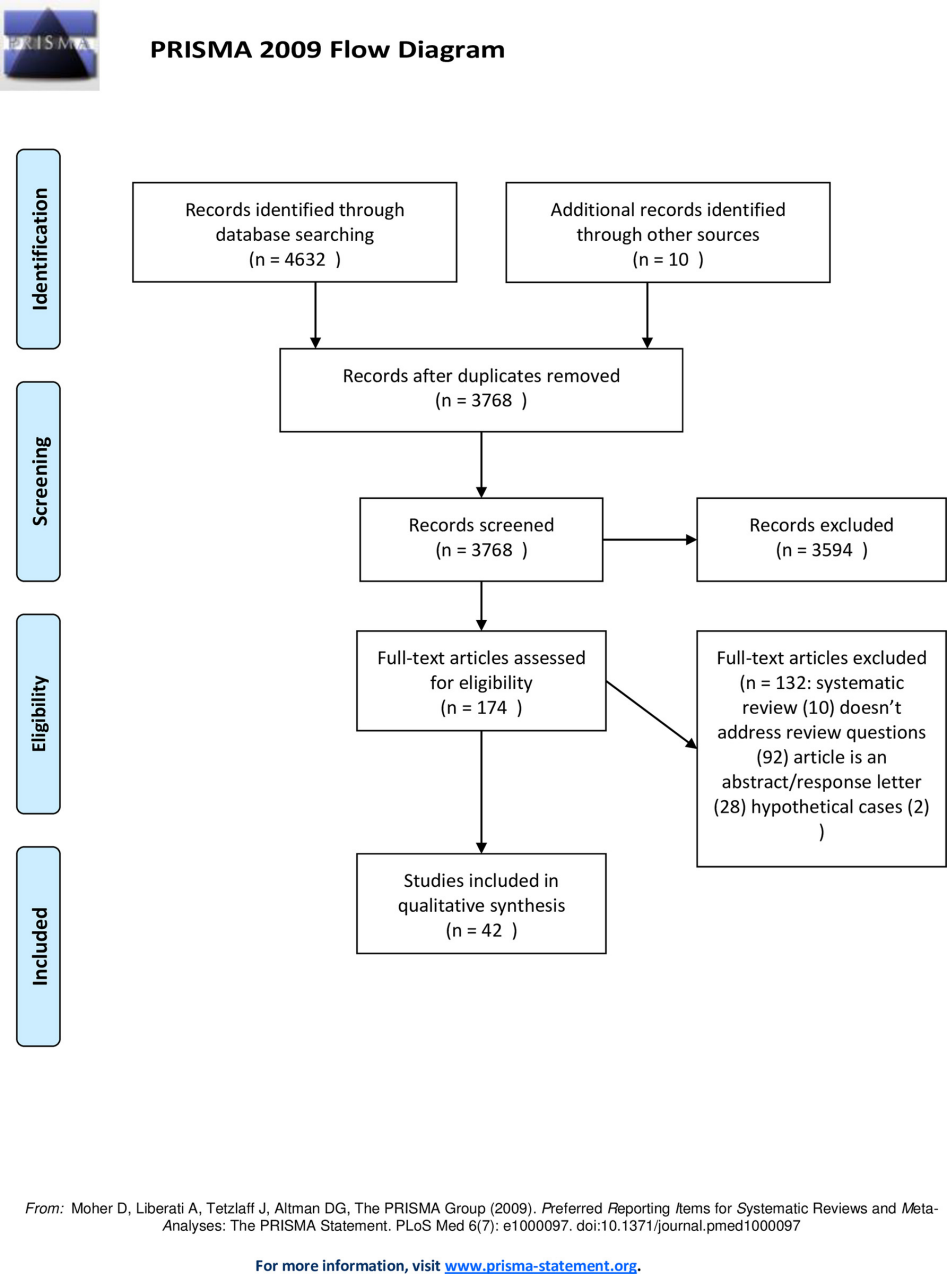
PRISMA study flowchart.

A summary of the review process is shown in Fig 1. A total of 4,642 records were identified; 4,632 from databases and 10 from a search of references. Of these, 874 were duplicates and 3,594 were excluded after screening their abstract/title. We retrieved 174 papers for appraisal of which 132 were subsequently excluded (S3 Appendix) and 42 studies were included in this review [19–60]. No unpublished studies were identified.

All of the studies addressed the question regarding clinician accuracy, and 17 studies included information that addressed the question about which clinicians were more accurate at prognosticating than others (Table 1). The participants of 25 (58%) studies had cancer, one (2%) study concerned participants with liver disease, and 17 (40%) studies contained both patients with cancer and non-cancer diagnoses. To assess reliability of the quality assessment, every second paper (alphabetically) included in the review was also scored for quality by the second reviewer, with moderate agreement, k = .6334, *p* < .001 [61]. The patient population, prognostic factor, outcome, and statistic domains were generally at low risk of bias across the studies. The clinician population and attrition domains had moderate levels of bias. The risk of bias due to confounding variables was moderate to high (S4 Appendix).

**Table 1:** Papers included in the review.

First Author	Disease	Number of estimates	How accurate are clinicians?			Are some clinicians more accurate than others?	
			Categorical	Continuous	Probabilistic	Categorical	Continuous
Addington-Hall [19]	Any	1128	X			X	
Brandt [40]	Any	511	X				
Bruera [20]	Cancer	94	X			X	
Buchan [41]	Any	13	X				
Casarett [42]	Any	21074	†				
Fromme [45]	Any	429	X				
Glare [46]	Cancer	44	X				
Glare & Virik [34]	Any	100	X				
Gripp [25]	Cancer	580	X			X	
Gwilliam [47]	Cancer	987±	X			X	
Holmebakk [26]	Cancer	243	X			X	
Kao [49]	Cancer	50	X				
Llobera [27]	Cancer	600	X			X	
Muers [54]	Cancer	203	X	X			
Selby [36]	Any	36	X				
Shah [55]	Any	248	X				
Stiel [56]	Any	82	X				
Twomey [30]	Any	126	†			†	
Vigano [58]	Cancer	233	X			X	
Zibelman [60]	Any	273	X				
Thomas [37]	Multiple**	254	X				
Chow [21]	Cancer	739		X			X
Chritakis [22]	Any	468		X			X
Evans [33]	Cancer	149		X			
Faris [44]	Cancer	162		X			
Forster [24]	Any	540		X			X
Heyse-Moore [38]	Cancer	50		X			X
Higginson [48]	Cancer*	275		X			
Lamont [51]	Cancer	300		X			
Maltoni, ‘94 [31]	Cancer	100		X			X
Maltoni, ‘95 [39]	Cancer	530		X			
Morita [53]	Cancer	150		†			
Oxenham [28]	Any	30		X			X
Parkes [29]	Cancer	74		X		X	
Lam [50]	Cancer	167		X			
Mackillop [52]	Cancer	39		†			
Taniyama [57]	Cancer	75		X			
Fairchild [23]	Cancer	395	X	X		†	X
Hui [35]	Cancer	127	X	X	X	X	X
Cooper [43]	Liver Disease	456			X		
Knaus [32]	Any	4028			X		
Weeks [59]	Cancer	917			X		

*was originally all diseases but only cancer patients included in the analysis

**Cancer, COPD, Heart Failure

† Not included in analysis, narratively described ± Estimates from MDT data only

### How accurate are clinician predictions of survival in palliative care patients?

Of the 42 studies included, 20 reported prognostic estimates using only a categorical approach [19, 20, 25–27, 30, 34, 36, 37, 40–42, 45–47, 49, 55, 56, 58, 60], 16 reported only continuous estimates [21, 22, 24, 28, 29, 31, 33, 38, 39, 44, 48, 50–53, 57] and 3 studies reported only probabilistic estimates [32, 43, 59]. Two studies used both categorical and continuous estimates [23, 54] and one study reported all three types of estimates [35].

#### Studies reporting categorical prognostic estimates

The papers varied widely in regards to the number of prognostic categories and the boundaries for each category. Some studies reported clinicians’ predictions about whether patients would survive to a particular time point (e.g. greater or less than 4 weeks) and others consisted of multiple categories (e.g. “days”, “weeks”, “months” or “years”) (Table 2). In some studies clinicians were asked an open survival question (i.e. continuous), but the data were subsequently reported categorically as either “accurate” (which contained an upper and lower threshold for inclusion of the category, such as ±33%), “under estimate”, or “overestimate”.

**Table 2:** Summary of studies in which clinicians were asked to predict survival using defined categories (categorical studies).

First Author	Number of categories	Description of categories
Addington-hall, 1990	2	< or > 1 year
Bruera, 1992	2	< or > 4 weeks
Buchan, 1995	2	Is death imminent? (yes/no)
Casarett, 2012	2	Is death imminent? (yes/no)
Shah, 2006	2	“Good prognoses” (> 1 year) and “Poor prognoses” (< 12 months)
Brandt, 2006	3	Within 1 week (0–7 days); death within 1–3 weeks (8–21 days); and death within 4–6 weeks (22–42 days).
Gripp, 2007	3	< 1 month; 1–6 months; > 6 months
Muers, 1994	3	< 3 months; 3–9 months; >9 months
Vigano,1999	3	< 2 months; 2–6 months; >6 months
Gwilliam, 2013	3	‘Days’ (< 14 days); ‘Weeks’ (2 weeks to less than 8 weeks); ‘Months or Years’ (≥ 2 months).
Fromme, 2010	4	<3 days; 4 days to 1 month; >1 month to 6 months; >6 months.
Fairchild, 2014	4	Days; Weeks; Months; Years
Llobera, 2000	4	< 30 days; 31–90 days; 91–180 days; > 180 days
Kao, 2011	5	Weeks; Months; 1 year; < 2 years, > 2 years
Zibelman, 2014	5	Hours–Days: < 4 days; Days–Weeks: 4–30 days; Weeks–Months: 31–180 days; Months–Years: >181 days; Nonspecific or no time frame given
Glare, 2004	6	If prognosis was believed to be < 3 months; then asked to express the prognosis in 2-week intervals, up to a maximum of 12 weeks
Glare, 2001	6	1–2 weeks; 3–4 weeks; 5–6 weeks; 7–10 weeks; 11–12 weeks; > 12 weeks.
Twomey, 2008	6	< 24 hours; > 24 hours but < 72 hours; > 72 hours but < 10 days; > 10 days but < one month; > one month but < three months; > three months
Stiel, 2010	7	1–2 weeks, 3–4 weeks, 5–6 weeks, 7–8 weeks, 9–10 weeks, 11–12 weeks, > 12 weeks
Hui, 2011^†^	7	24 hours; 48 hours; 1 week; 2 weeks; 1 month; 3 months; 6 months.
Selby, 2011	7	< 24 hours; 1–7 days; 1–4 weeks; 1–3 months; 3–6 months; 6–12 months; > 12 months
Thomas, 2009	7	< 1 month; 1–6 months; 7–12 months; 13–23 months; 2–5 years; 6–10 years; > 10 years
Holmebakk, 2011	8	< 1 week; 1–4 weeks; 1–3 months; 3–6 months; 6–9 months; 9–12 months; 12–18 months; 18–24 months

^†^ This study appears in other table

**Fig 2:**
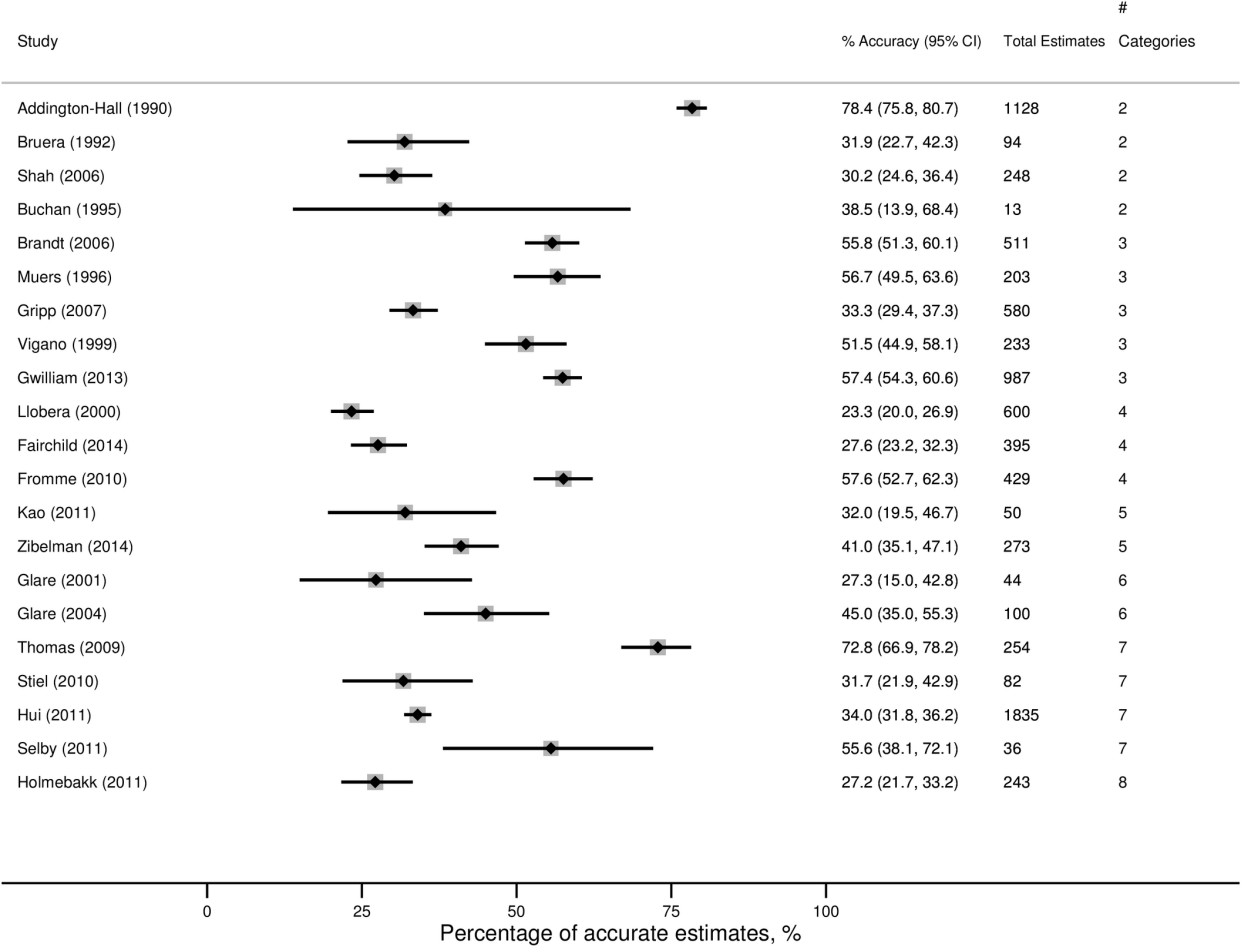
Summary data from studies in which clinicians provided categorical survival estimates (grouped by number of categories). The data represented is the percentage of accurate estimates given out of the total number of estimates given. Note: The study by Gwilliam et al (2013) included doctor, nurse and MDT estimates. However, since the estimates were not independent of each other, only the MDT estimates have been presented here.

The accuracy of the categorical prognostic estimates in the 21 studies for which percentage accuracy could be calculated are presented in Fig 2 [19, 20, 23, 25–27, 34–37, 40, 41, 45–47, 49, 54–56, 58, 60]. Two papers could not be included in the forest plot because the relevant data was not available. Twomey, O’Leary, & O’Brien [30] reported that clinicians were just as likely to overestimate as to underestimate survival. Casarett, Farrington, & Craig *et al* [42] reported the *c*-statistic for the accuracy of predicting death between one and 10 days. They reported that the c-statistic varied between a minimum of 0.61 (14 day survival) to a maximum of 0.72 (7 day survival). A *c*-statistic of 0.7 or higher is generally considered acceptable evidence of ability to discriminate [62].

The included studies reported 8,338 prognostic estimates. Fig 2 shows the variation in accuracy between the studies (range 23% to 78%). In one study [19] with only two prognostic categories, accuracy was reported as 78% (96% CI 75.8, 80.7); in contrast another study [20] also with only two prognostic categories, reported accuracy at 32% (95% CI 22.7, 42.3). Similarly with seven prognostic categories to choose from, one study [37] reported accuracy as 73% (95% CI 66.9, 78.2) and another study [35] reported accuracy as 34% (95% CI 31.8, 36.2). Possible reasons for this spread of results include variation in the number of pre-defined categories between the studies, the diverse nature of the clinical populations (age, diagnoses, gender mix), the characteristics of the clinicians, and the setting for the studies (hospice, community or hospital). Eight studies [19, 20, 23, 25–27, 35, 47] reported estimates from more than one type of clinician, typically doctors and nurses. Thirteen papers reported the estimates of a single group of clinicians [34, 36, 37, 40, 41, 45, 46, 49, 54–56, 58, 60]. Nine of these studies reported the accuracy of the medical profession [34, 37, 40, 49, 54–56, 58, 60]; three studies reported the accuracy of a multidisciplinary team [36, 45, 46]; and one study [41] reported the accuracy of nurses. (S5 Appendix).

#### Studies reporting continuous prognostic estimates

Table 3 shows data from 17 studies involving 4,511 continuous prognostic estimates [21–24, 28, 29, 31, 33, 35, 38, 39, 44, 48, 50, 51, 54, 57]. As with the categorical data the results from these studies were very heterogeneous. The studies show that predicted median survival ranged from 14 to 219 days and actual median survival ranged from 10 to 126 days. The difference between median predicted and median actual survival ranged from an underestimate of 86 days to an overestimate of 93 days. In five of the studies the median difference showed an underestimate, while thirteen showed an overestimate.

**Table 3:** Predicted versus actual survival in those studies where clinicians were asked to provide a continuous temporal estimate of survival (continuous studies).

First Author	Number of prognostic Estimates	Predicted survival in days (median)	IQR	Actual survival in days (median)	IQR	Difference between predicted and actual survival
Chow, 2005	739	25	nr	111	nr	-86
Christakis, 2000	468	77	28–133†	24†	12–58†	53
Evans, 1985	149	81	28–182	21	43–180	60
Fairchild, 2014	395	219	nr	126	nr	93
Faris, 2003	162	21	45–135	10	nr	11
Forster, 1988	540	46	nr	24	nr	22
Heyse-Moore, 1987	50	56	33–84†	14	7–28†	42
Lam, 2008	167	70	43–137	76	30–160	-6
Lamont, 2001	300	75	nr	26	nr	49
Maltoni, 1994	100	42	nr	35	nr	7
Maltoni, 1995	530	42†	28–70†	32	13–62†	10
Muers, 1996	203	36	21–82	38	22–85	-2
Oxenham, 1998	30	21†	14–35†	17	9–25†	4
Parkes, 1972	74	28†	24–56†	21†	9–34†	7
Taniyama, 2014	75	120	60–180	121	40–234	-1
Higginson a, 2002	275	28	10–32	42	nr	-14
Higginson b, 2002		84	176–516		nr	42
Hui a, 2011	127	14	nr	12	nr	2
Hui b, 2011^†^	127	20	nr	12	nr	8

Higginson a: upper estimate Higginson b: lower estimate.

Hui a: Doctors, Hui b: Nurse

**†** Data extracted from previous review [11]

nr: not reported

IQR: Interquartile Range

^†^ This study appears in other tables

**Fig 3:**
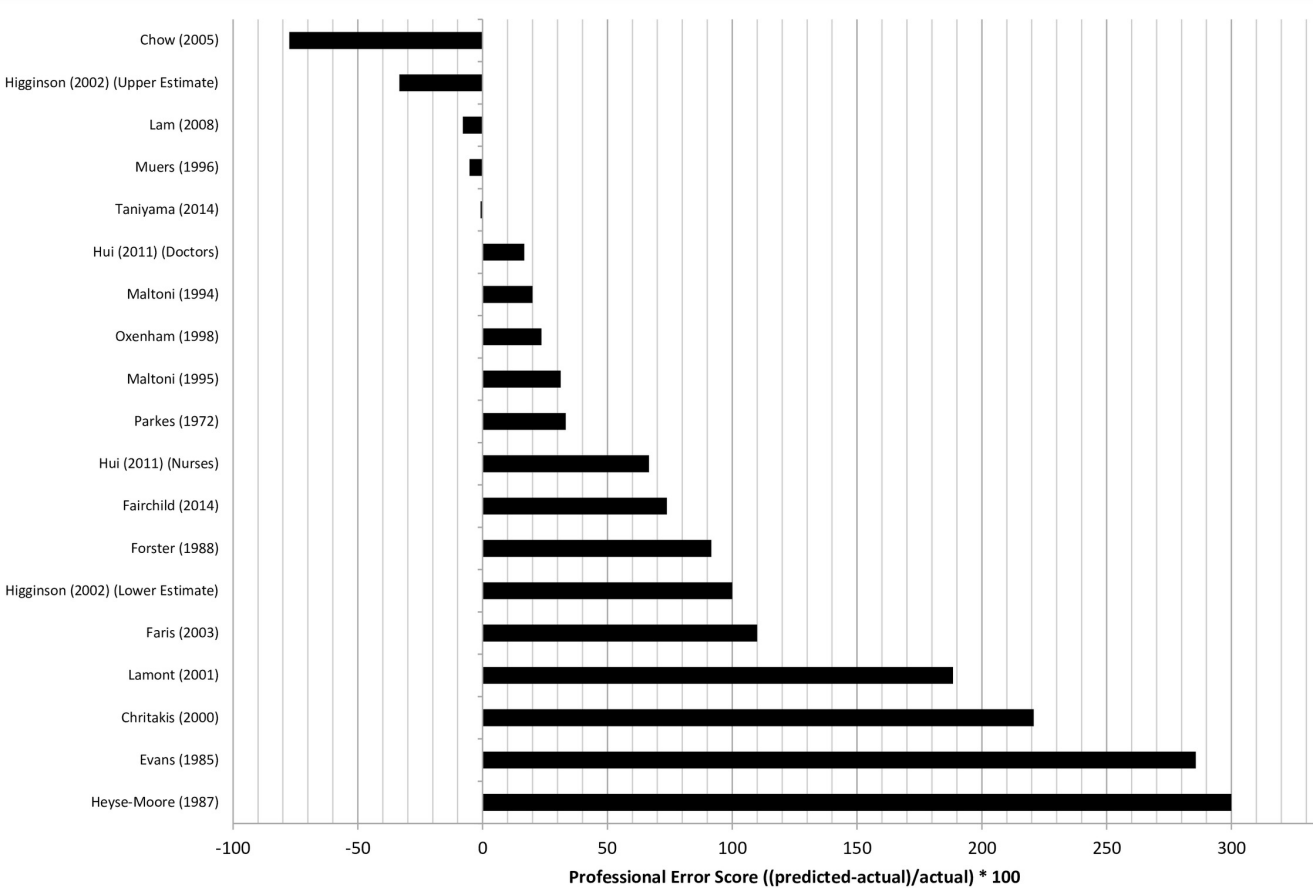
Professional Error Score (PES) of clinicians’ estimates of survival those studies where clinicians were asked to provide a continuous temporal estimate of survival. The black bar in this figure indicates the overall accuracy of the clinicians’ estimates (0 indicates perfect accuracy, positive values indicate over-estimates and negative values indicate under-estimates).

Fig 3 shows the professional error score for each study. Two of the studies [35, 48] in Fig 3 appear twice as each study reported two separate prognostic estimates: from doctors and nurses in one paper [35] and upper and lower estimates in another [48].

Mackillop & Quirk [43] reported that doctors have an acceptable ability at predicting survival of three months (ROC value = 0.75, ±0.04 SE), but they are only slightly better than a random guess at 1 year (0.57, ±0.01 SE). Morita, Tsunoda, & Inoue *et al* [46] reported the results of two studies. The first study evaluated clinical predictions of survival by palliative care physicians and reported a moderate correlation between estimated and actual survival (r = 0.62). The second study evaluated clinical predictions of survival by palliative care physicians, with the aid of a prognostic tool, and reported a strong correlation between estimated and actual survival (r = 0.74).

#### Studies reporting probabilistic prognostic estimates

Four papers used a probabilistic scale as a measure of accuracy [32, 35, 43, 59]. Hui, Kilgore, & Nguyen *et al* [35] asked doctors and nurses, “What is the approximate probability that this patient will be alive (0%–100%)?” for ≤24 hours, 48 hours, 1 week, 2 weeks, 1 month, 3 months, and 6 months. If a patient survived and the clinician had a survival percentage prediction of ≥70%, or the patient died within the time frame and the clinician had a survival prediction of ≤30%, they were considered correct estimates. Their results indicated that probabilistic prognostic estimates were more accurate than a continuous approach, for each time frame (*p* = < .001 for all paired comparisons). The other three [32, 43, 59] studies asked clinicians what was the percentage likelihood of six month survival and reported a ROC value between 0.74–0.78, which can be interpreted as demonstrating an acceptable level of accuracy.

### Are any subsets of clinicians more “expert” at prognosticating than others?

In total, 17 studies were identified which addressed the issue of which group of clinicians are more accurate than others [19–31, 35, 38, 47, 58].

#### Clinician characteristics

Nine papers provided only minimal details about the clinicians (e.g. job title or specialty) [19, 23, 24, 27–30, 38, 58]. Five papers reported the experience of the clinician; either in years, or narratively [20, 21, 25, 26, 31]. Bruera, Miller, & Kuehn *et al* [20] studied two clinicians who were described as “highly experienced”. Three papers provided more detailed characteristics about the clinicians [22, 35, 63] (Table 4).

**Table 4:** Summary of studies investigating differences in prognostic accuracy between clinical groups.

First Author	Estimate	Professional groups	Description of clinician-level factors	Main findings
Addington-Hall (1990)	Categorical	Doctors; Nurses	Job title	No difference
Bruera (1992)	Categorical	Doctors	2 Doctors “highly experienced and dedicated to full time management of patients with advanced cancer”	No difference
Chow (2005)	Continuous	Radiation Oncologists	Years of experience	No difference; inaccurate and tended to be overly optimistic.
Christakis (2000)	Continuous	Doctors	Job title; Self-rated optimism; years of experience; gender; board certified; length of time known patient; contact time with patient; number of referrals to hospice	Overall, not very accurate. Experience decreases risk of optimistic and pessimistic errors.
Fairchild (2014)	Both	Doctors; Radiation therapist; Nurses; Allied health professionals	Job title	Radiation therapists more accurate than allied health professionals.
Forster (1988)	Continuous	Consulting oncologist; General Internist; Hospice social worker; Community oncologist; Nurse	Job title	Registered nurse and consulting university oncologist were more accurate but still overly optimistic
Gripp (2007)	Categorical	Doctors; Experienced Physician; Tumour Board	Years of experience	No difference.
Gwilliam (2013)	Categorical	Doctors; Nurses; MDTs	Age; Gender; Length of time qualified; Length of time working in palliative care; Time known patient; Time since last assessed patient	No difference between doctors’ and nurses’ accuracy. MDTs more accurate than a nurse alone. Nurses’ accuracy better when patient reviewed within previous 24 hours
Heyse-Moore (1987)	Continuous	Hospital Doctors; GPs	Job title	No inferences made about groups in paper, but data shows GP slightly better
Holmebakk (2011)	Categorical	Surgeons	Years of experience	No difference.
Hui (2011)	Both	Doctors; Nurses	Age; Gender; Ethnicity; Religion; Years of experience; Years of palliative experience	With probabilistic prediction, nurses more accurate with 24 hour and 48 hour time points.
Llobera (2000)	Categorical	Oncologists; Nurses; GP	Job title	Oncologists and nurses are more accurate than GP
Maltoni (1994)	Continuous	Oncologists	Years of Experience	The more experienced oncologists were more accurate
Oxenham (1998)	Continuous	Doctor; Sister; Staff Nurse; Chaplain; Auxiliary	Job title	Auxiliary most accurate with imminent death
Parkes (1972)	Categorical	Referring Doctor; Referring GP; Doctors; Nurses.	Job title	No difference.
Twomey (2008)	Categorical	Consulting university oncologist; General Internist Hospice social worker; Community oncologist; Registered Nurse	Job title	No group accurately predicted the length of patient survival more than 50% of the time. Nursing and junior medical staff were most accurate while care assistants were least accurate. When in error, senior clinical staff tended to under estimate survival.
Vigano (1999)	Categorical	Oncologists	Job title	No difference

Christakis & Lamont [22] reported clinicians’ individual characteristics: job title, self-rated optimism, experience, gender, board certification. They reported the doctor-patient relationship: how long they had known the patient, how frequently they had seen the patient, and the last time they saw the patient. They reported how many times they had referred someone to a hospice in the last year and how many patients they had met with a similar diagnosis. Gwilliam, Keeley & Todd *et al* [47] reported the following clinician characteristics: age; gender; length of time qualified; length of time working in palliative care how long they had known the patient and when they had last assessed them. Hui, Kilgore, & Nguyen *et al* [35] reported the clinicians’ age, gender, ethnicity, religion and years of experience (overall and within palliative care).

### Overall differences in prognostic ability between clinicians

Overall, 7/17 (35%) studies found no difference in prognostic ability between different types of clinicians (however defined) [19–21, 25, 26, 29, 58] (Table 4).

Six studies identified a difference between the prognostic accuracy of different clinicians. Gwilliam, Keeley & Todd *et al* [47] reported that a multidisciplinary estimate was more accurate than a nurse or doctor individually. They also reported that accuracy was not affected by gender, age, grade, experience, or length of time that the clinician had known the patient. However, nurses who had assessed a patient within the last 24 hours were more accurate than nurses who had not seen the patient within that time frame (p<0.01). Fairchild, Debenham, & Danielson, *et al* [23] compared the accuracy of doctors, radiation therapists, nurses, and allied health professionals. They reported that, overall, there was no difference between the prognostic accuracy of these groups, but radiation therapists were more accurate than allied health professionals. Twomey, O'Leary, & O'Brien[30] studied oncologists with varying levels of experience, social workers, and nurses. They reported that overall accuracy was below 50%, but nurses and junior doctors were more accurate than care assistants, nurse managers, and consultants. Heyse-Moore & Johnson-Bell [38] reported the accuracy of referrals to a hospice from hospital doctors and general practitioners (GPs). Although no comparisons were reported in the study, the results suggest that GPs were more accurate at predicting survival than the other groups. Llobera, Esteva, & Rifa *et al* [17] studied oncologists’, nurses’, and GPs’ estimates. They report that oncologists and nurses were more accurate than GPs. Forster & Lynn [29] studied oncologists of different grades, social workers, and nurses. They found that, whilst oncologists were more accurate than the other groups, accuracy overall was still optimistic.

Two studies found that the time frame of the prognosis can impact the accuracy. Hui, Kilgore, & Nguyen *et al* [38] found that nurses are better at predicting imminent death, whereas doctors are better at predicting three and six month survival. Oxenham & Cornbleet [48] reported the accuracy of a hospice team, consisting of a doctor, a sister, a staff nurse, a chaplain, and auxiliary staff. The results from this study suggest that doctors are more accurate than other groups when asked to provide a prognostic estimate at the initial assessment, but that auxiliary staff members are better at predicting when a patient’s death is imminent.

Two studies reported that experience can lessen prognostic errors. Christakis & Lamont [22] studied doctors’ estimates, documenting the characteristics of the doctors. The results suggested that overall, accuracy was low, but that length of experience may decrease the risk of errors (both over estimates and under estimates), as the more experienced doctors were less likely to make an error. Maltoni, Priovano, & Scarpi *et al* [31] reported that more experienced oncologists were more accurate at prognosticating.

## Discussion

This systematic review identified 42 papers, spanning almost 30 years of research, and providing data on over 12,000 prognostic estimates. When clinicians were asked to provide a prognostic estimate from a pre-defined list of outcomes, accuracy varied from 23% to 78%. The clinical heterogeneity of the studies made it inadvisable to calculate an overall accuracy score. A previous systematic review [11] calculated an overall pooled accuracy score despite the high level of observed heterogeneity. Applying the same approach to our results would have indicated that clinicians over estimated survival by a factor of approximately two (44 days median predicted survival, 25 days median actual survival). However, for the reasons previously stated, this result should be viewed with caution. Although only recorded in four papers the evidence suggests that probabilistic estimates may be slightly more accurate than categorical or continuous temporal estimates of survival. There was no consistent evidence that one professional group or sub-group of clinicians was any more accurate than any other profession or sub-group. The level of experience of the clinician in some studies [31, 42] was seen as a factor that improves accuracy; however this was not replicated in all studies [20, 21, 25]. The time frame of the prognosis (e.g. prediction of imminent death versus prediction of death within 12 months) appeared to affect both the accuracy overall and the relative accuracy of different professionals [26, 35]. Some of the studies suggested that nurses and healthcare assistants are better at recognising imminent death than other professionals [28, 35]. Finally, two studies suggested that accuracy is better when the prognosis is made by a multidisciplinary team rather than by an individual clinician [25, 47].

### Strengths and Limitations

This is the first systematic review which has analysed the accuracy of prognostic estimates of all diagnoses, according to the type of estimate (categorical, continuous or probabilistic), and the characteristics of the clinician (e.g. professional group, years of experience).

Cochrane recommends that ideally two independent reviewers should extract data from identified papers during a systematic review [61]. In this review, only one reviewer extracted data which could introduce bias to the results. However, we feel that the potential for bias that this introduced has been limited through the use of a standardised extraction table which specified which information, agreed by all authors, was to be extracted in order to address the review questions.

It was challenging to find a comprehensive search strategy to identify all relevant studies for inclusion in this review. In order to ensure that we identified as many relevant studies as possible, the specificity of our search strategy was relatively low and hence a large number of studies were initially identified. Even with a low specificity search strategy, ten of the studies included in this systematic review were only identified during the hand search of references, which raises the possibility that our search may have not identified all potential studies. We wanted to identify only those studies where clinicians specifically quantified the prognosis (in terms of duration or probability of survival). The Gold Standards Framework is an approach to optimising care for patients approaching the end of life and is widely used in general practice, care homes and hospitals [64]. As part of the GSF approach clinicians are encouraged to identify those patients about whom they would not be “surprised” if they died within the next year. Although this screening question is quite widely used for identification of patients approaching the end of life, we did not include any studies evaluating this approach in our review because it does not require clinicians to estimate how long a patient is expected to live, nor to gage the probability that they will die within the next year.

Due to the degree of clinical heterogeneity among included studies, it was not possible to conduct a meta-analysis to provide a pooled estimate of accuracy. Studies were clustered by the type of prognostic estimate that was obtained (categorical, continuous or probabilistic). However, even within these subgroups, categorical and continuous studies were still highly heterogeneous. Diverse outcome measures, missing data, and limited information on the demographics of the clinicians made the accuracy of categorical estimates and the question about which clinicians are better prognosticators difficult to address. Details about the clinicians being asked to provide a prognosis in the included studies were often limited. Additional information about clinicians (beyond simply reporting their profession) was only provided in 8/17 (47%) studies. This lack of information limited our analysis about the factors which distinguished more “expert” prognosticators from those less accomplished in this clinical skill.

The appraisal of the quality of each study was challenging. The method of appraising prognostic studies is currently under development by the Cochrane Prognosis Group. The QUIPS tool is suggested by the Cochrane group as a suitable risk of bias instrument; however some of the areas covered by the tool were not always relevant to this systematic review (e.g. the concept of attrition). The accuracy of survival estimates, particularly when considered by profession of clinician, was often a secondary analysis in the included papers rather than being the primary outcome. Any systematic review is only as good as its included studies. Our assessment of the quality of individual studies suggested moderate to high risk of bias due to confounding factors; however most of the domains assessed were rated as having low risk of bias.

### Future implications of this review

Accurate prognoses are recognised as being of clinical importance for patients at all stages of the palliative care trajectory, from those recently referred to palliative care services [63] to those patients approaching the end of life [65]. Accuracy of categorical estimates in this systematic review ranged from 23% up to 78% and continuous estimates over-predicted actual survival by, potentially, a factor of two. This systematic review highlights the heterogeneous nature of studies of prognostic accuracy in palliative care. Future research, to potentially reduce the heterogeneity and increase accuracy, could be to incorporate a validated prognostic tool [53], using agreed “clinically relevant” prognostic categories. Examples of such tools include the Prognosis in Palliative Care predictor models[63] and the Palliative Prognostic score [66]. Alternatively, a recent review article [67], highlights that treatment plans and decisions can be made without such a weighted focus on survival estimates.

The Neuberger review into the use of the LCP [9] recommended that evidence-based education and competency-based training should be promoted to improve prognostic skills. However no clear guidance exists on how clinicians can be taught to perform this task better. There are currently no evidence-based education programmes to train clinicians how to become better prognosticators. Some studies suggest that more experienced or better qualified clinicians or different members of the MDT may be better than others at making prognostic predictions. Future research should try to identify how these clinicians have become better prognosticators so that evidence-based training can be developed for their less accurate colleagues.

## Supporting Information

S1 Appendix Systematic Review Protocol. (DOCX)

S2 Appendix Search Strategy. This is the strategy that was employed on the OVID platform and modified for other databases.
(DOCX)

S3 Appendix Breakdown of full study exclusion reasons. (XLSX)

S4 Appendix QUIPS risk assessment scores. (DOCX)

S5 Appendix Data used to complete the analysis of categorical estimates. (XLSX)
