# Supplementary material for: A Systematic Review of Predictions of Survival in Palliative Care: How Accurate Are Clinicians and Who Are the Experts?
Source: PLoS One. 2016 Aug 25;11(8):e0161407. doi: 10.1371/journal.pone.0161407 (PMC4999179; doi:10.1371/journal.pone.0161407)
Supplement: S1 Appendix — (DOCX) [file pone.0161407.s001.docx]

Systematic Review Template

**Title of Review:**

How accurate are clinicians at formulating a prognosis in a palliative population?

**Reviewer contact details:**

Name: Nicola White

Email: [n.g.white@ucl.ac.uk](mailto:n.g.white@ucl.ac.uk)

Telephone: 0207 679 9057

**Background:**

According to the report “Deaths in Older adults in England” (2010) there are currently 4.0 million people aged 75 and over. This is projected to increase to 7.2 million in the year 2033. The National End of Life Strategy (2008) aims to get health professionals to identify individuals in the last year of their life in order to prepare for the eventual event of death through an Advance Care Plan. By doing this, it ensures that the patient’s wishes are followed as well as keeping costs for unnecessary interventions down. There have been recent reports that indicate people do not always die at the location of their choice, and that palliative care services can help achieve this goal. ^[[1]](#endnote-1),^^[[2]](#endnote-2)^

The crux of prognosis is the accurate recognition of death by health care professionals. The National End of Life Care Intelligence Network published a report ‘Predicting Death’ which looked in to expected deaths in England and Wales (2011); comparing several reports, the ‘unexpected death’ figure lay between 22% - 42%.

For those recognised as dying, the Liverpool Care Pathway (LCP) was first introduced as a caring model for symptom control^[[3]](#endnote-3)^. It was one of three tools recommended as part of the National Institute for Health and Clinical Excellence guidelines (2004) for promoting high quality end-of-life care. The recent independent report commissioned on the LCP (“More care, Less Pathway”, 2013^[[4]](#endnote-4)^) has highlighted how imprecise the diagnosis of dying is. The same report also noted how people were placed on the pathway when they were not imminently dying, therefore not receiving the appropriate care. This emphasizes the need for research in this area.

A report by Parry, Seymour, Whittaker, Bird, & Cox (2013)^[[5]](#endnote-5)^ stated that there is a lack of research in to the area of prognosis and imminent death which would help healthcare professionals when faced with these difficult and emotive situations.

**Clarification of research questions and scope**

Systematic Reviews within prognosis is a relatively new field with limited guidance on how to complete a thorough search. The standard PICOT guidelines do not provide the best template for this sort of review. The Prognosis Methods Group as part of the Cochrane Collaboration^[[6]](#endnote-6)^ set out the following guidance for reviewing prognosis study:

1. What is the course of the condition/disease? (Descriptive)

2. What prognostic factors are associated with outcome? (Explanatory)

3. What groups of prognostic factors best predict outcome? (Outcome prediction)

4. What are the interactions between intervention and prognostic factors?

This systematic review will focus on addressing the second and third part of this guidance; whereby the outcome will be mortality.

As indicated by Altman (2001)^[[7]](#endnote-7)^, prognostic studies are usually difficult to search for and are often found to be poor methodologically. In this paper, they gave systematic search terms that provide ‘*the best complex search strategy with the highest sensitivity’* to identify prognosis studies. These search terms have been adopted in this systematic review.

The overall aim of the review is to understand how accurate clinicians are at providing a prognosis within a palliative population. Secondary to that, the review will also review if any particular subgroups of clinicians are more accurate than others and to see how clinicians are formulating their prognoses.

The review will look at all study designs in which prognosis and survival estimates are addressed.

Studies that look at survival following an intervention or admission to ICU or children will not be looked at in this review. This is because ICU prognosis is in relation to the intervention or withdrawal of intervention which would confound the factors we wish to look at. Research focused on paediatric research, it could be hypothesized, are unlikely to have the same prognostic factors. Animal studies will also not be included in the search.

**Focused Review Question**

How accurate are clinicians at formulating a prognosis in a palliative population?

*More specifically, the review aims to:*

1. Examine how accurate clinicians are at providing a prognosis within a palliative population
2. Examine which subgroups of clinicians are more accurate than others with their prognosis
3. Examine how clinicians are formulating their prognosis

**Inclusion and Exclusion Criteria**

| **Selection Criteria** | **Inclusion** | **Exclusion** |
| --- | --- | --- |
| Population | Adults (defined as age >18 years)  Palliative ; Terminal | **NOT** ICU or receiving artificial ventilation or advanced life support  **NOT** Paediatric population  **NOT** animal studies |
| Population | Clinicians within a palliative setting who have given an estimate of survival / prognosis |  |
| Prognosis | Any study which addresses prognosis and mortality | **NOT** prognosis following an intervention (e.g. surgical intervention) |
|  |  | **NOT** if there is a lack of clarity on prognosis or timeframe of mortality  **NOT** if the prognostic factors would not be routinely available in clinical practice  **NOT** if study is assessed as high risk of bias |
| Study Design | Any study design which includes prognosis | |
| Language | English | |
| Other | Full papers only | |

**Search Strategy**

| **Selection Criteria** | **Categories** | **Potential Search Terms** |
| --- | --- | --- |
| Population | Adults (defined as age >18 years)  Palliative ; Terminal | Population in receipt of palliative care or in the end of life  Population in any setting: hospital, community, hospice. |
| Population | Clinicians’ prediction | Clinician ; Physician; Nurse; Doctor; Healthcare professional  **and**  Estimate of survival; Prediction; Life expectancy |
| Prognosis | Search terms to find prognostic studies | Prognosis as suggested by Altman  Incidence; Mortality; Follow-up studies; Mortality; Prognosis; Predict; Course  **Other:**  Survival analysis |

**Sources to be searched**

The following databases will be searched from 1946 onwards: Medline, Embase, CINAHL, AMED, Science Citation Index, Cochrane Database of Systematic Reviews, Cochrane Central Register of Controlled Trials. The reference lists of included studies and relevant review articles will also be checked.

**Search terms**

Developed in collaboration with Judith Scammell, Librarian, St George’s university.

**Study Selection**

Two reviewers will be involved throughout this process – the PhD student and the two supervisors taking the second role between then.

The PhD student will initially screen all citations that are identified from the search and remove any that are obviously irrelevant or duplicates, using the study selection criteria for abstracts (see appendix 2). The second reviewer will check the list of excluded articles to check validity. Any discrepancies will be discussed until a final consensus is reached.

The full text will be ordered for the remaining articles and go through the study selection criteria for full texts (see appendix 3). This will be piloted on the first 5 papers to check its sensitivity. The articles will be reviewed independently by the second reviewers to check inter-rater reliability. As done at the initial stage, any discrepancies will be discussed until a final consensus is reached.

If there is missing data needed to decide inclusion, the authors of the article will be contacted.

**Data Extraction**

The full texts that are included in to the analysis will then have the data extracted from them by the PhD student using a standardised data extraction form based on the Cochrane Review handbook (see appendix 4). It will be piloted on the first 3 papers for suitability to a prognosis study. Data extraction will be checked by a second reviewer through random sampling. Studies that give rise to uncertainty will be reviewed by a second researcher, and any disagreements will be resolved by discussion.

**Quality Assessment Strategy**

Risk of Bias will be assessed using the QUIPS tool designed by Hayden et al (2013)^[[8]](#endnote-8)^ which has been adapted specifically for prognostic studies. See appendix 1.

**Proposed Data synthesis**

The precise method of analysis and synthesis will be determined by the availability, volume, and homogeneity of the studies identified.

**Appendix 1 QUIPS tool (Hayden *et al,* 2013)**

| **Biases** |
| --- |
| Instructions to assess the risk of each potential bias: |
| **1. Study Participation** |
| Source of target population |
| Method used to identify population |
| Recruitment period |
| Place of recruitment |
| Inclusion and exclusion criteria |
| Adequate study participation |
| Baseline characteristics |
| **Summary Study participation** |
| **2. Study Attrition** |
| Proportion of baseline sample available for analysis |
| Attempts to collect information on participants who dropped out |
| Reasons and potential impact of subjects lost to follow-up |
| Outcome and prognostic factor information on those lost to follow-up |
|  |
| **Study Attrition Summary** |
| **3. Prognostic Factor Measurement** |
| Definition of the PF |
| Valid and Reliable Measurement of PF |
|  |
| Method and Setting of PF Measurement |
| Proportion of data on PF available for analysis |
| Method used for missing data |
| **PF Measurement Summary** |
| **4. Outcome Measurement** |
| Definition of the Outcome |
| Valid and Reliable Measurement of Outcome |
| Method and Setting of Outcome Measurement |
| **Outcome Measurement Summary** |
| **5. Study Confounding** |
| Important Confounders Measured |
| Definition of the confounding factor |
| Valid and Reliable Measurement of Confounders |
| Method and Setting of Confounding Measurement |
| Method used for missing data |
| Appropriate Accounting for Confounding |
|  |
| **Study Confounding Summary** |
| **6. Statistical Analysis and Reporting** |
| Presentation of analytical strategy |
| Model development strategy |
|  |
| Reporting of results |
| **Statistical Analysis and Presentation Summary** |

**Appendix 2 Criteria for selecting studies at abstracts stage**

Primary Question: How accurate are clinicians at formulating a prognosis in a palliative population?

Specifically:

1. Examine how accurate clinicians are at providing a prognosis within a palliative population
2. Examine which subgroups of clinicians are more accurate than others with their prognosis
3. Examine how clinicians are formulating their prognosis

Include abstracts if:

- Study looks at prognosis
- Study includes clinicians estimates of survival
- Mortality is measured

Study types:

- RCT
- Cohort studies (retrospective and prospective)
- Unclear study design
- Case studies

Exclusions:

- Paediatric population or animal study
- Mechanical ventilation/ICU admission
- Prognosis is following an intervention (e.g. surgical)
- Not in English

**Appendix 3 Criteria for selecting studies at full text stage**

Primary Question: How accurate are clinicians at formulating a prognosis in a palliative population?

More specifically:

1. Examine how accurate clinicians are at providing a prognosis within a palliative population
2. Examine which subgroups of clinicians are more accurate than others with their prognosis
3. Examine how clinicians are formulating their prognosis

Include if:

- Study looks at: clinicians estimates of prognosis
- Study looks at subgroups of clinicians and the estimates provided
- Study addresses how clinicians formulate a prognosis
- Mortality is the focus of the prognosis in the three points above

Exclusions if:

- Mechanical ventilation/ICU admission
- Prognosis in response to an intervention

Study types:

- RCT
- Cohort studies (retrospective and prospective)
- Unclear study design
- Case studies/reviews/opinion pieces

Appropriate outcome measure used

- Death
- Prognosis

Exclusions

- Paediatric population or animal study
- Prognosis under 7 days is not addressed
- Not in English
- Duplicate study, only use data once

**FINAL DECISION (PLEASE TICK ONE)**

|  | INCLUDE |
| --- | --- |
|  | EXCLUDE, give reason using criteria above |
|  | NEEDS DISCUSSION IN RESEARCH GROUP, note points that require discussion overleaf |
|  | NEED TO CONTACT AUTHORS TO MAKE DECISION, note points overleaf that require clarification, also think ahead to data extraction phase |

**Appendix 4** Data Extraction tool (Cochrane Handbook Chapter 7 table 7.3a)

| **Source**   - Study ID (created by review author). - Report ID (created by review author). - Review author ID (created by review author). - Citation and contact details.   **Eligibility**   - Confirm eligibility for review. - Reason for exclusion.   **Methods**   - Study design. - Total study duration. - Sequence generation*. - Allocation sequence concealment*. - Blinding*. - Other concerns about bias*.   **Participants**   - Total number. - Setting. - Diagnostic criteria. - Age. - Sex. - Country. - [Co-morbidity]. - [Socio-demographics]. - [Ethnicity]. - [Date of study].   **Interventions**   - Total number of intervention groups.   *For each intervention and comparison group of interest*:   - Specific intervention. - Intervention details (sufficient for replication, if feasible). - [Integrity of intervention]. | **Outcomes**   - Outcomes and time points (i) collected; (ii) reported*.   *For each outcome of interest*:   - Outcome definition (with diagnostic criteria if relevant). - Unit of measurement (if relevant). - For scales: upper and lower limits, and whether high or low score is good.   **Results**   - Number of participants allocated to each intervention group.   *For each outcome of interest*:   - Sample size. - Missing participants*. - Summary data for each intervention group (e.g. 2×2 table for dichotomous data; means and SDs for continuous data). - [Estimate of effect with confidence interval; P value]. - [Subgroup analyses].   **Miscellaneous**   - Funding source. - Key conclusions of the study authors. - Miscellaneous comments from the study authors. - References to other relevant studies. - Correspondence required. - Miscellaneous comments by the review authors. |
| --- | --- |

1. The National Survey of Patient Activity Data for Specialist Palliative Care Services (2013) [↑](#endnote-ref-1)
2. The National Bereavement Survey (VOICES) ONS, 2013 [↑](#endnote-ref-2)
3. Ellershaw, J., & Ward, C. (2003). Care of the dying patient: the last hours or days of life. *BMJ: British Medical Journal*. Retrieved from http://www.ncbi.nlm.nih.gov/pmc/articles/pmc1124925/ [↑](#endnote-ref-3)
4. More care, less pathway: A review of the Liverpool Care Pathway. 2013. www.gov.uk/government/uploads/system/uploads/attachment_data/file/212450/Liverpool_Care_Pathway.pdf. (Last accessed 18^th^ November 2013). [↑](#endnote-ref-4)
5. Parry, R., Seymour, J., Whittaker, B., Bird, L., & Cox, K. (2013). *Rapid Evidence Review: Pathways Focused on the Dying Phase in End of Life Care and Their Key Components.* (pp. 1–35). Nottingham, United Kingdom. [↑](#endnote-ref-5)
6. http://prognosismethods.cochrane.org/ [↑](#endnote-ref-6)
7. Altman, D. Systematic reviews of evaluations of prognostic variables. BMJ. 2001 July 28; 323(7306): 224–228. [↑](#endnote-ref-7)
8. Hayden, J. (2013). Assessing bias in studies of prognostic factors. *Annals of internal; 158*(4), 280–286. Retrieved from http://annals.org/article.aspx?articleid=1650776&atab=10 [↑](#endnote-ref-8)
