# Supplementary material for: A Systematic Review of Predictions of Survival in Palliative Care: How Accurate Are Clinicians and Who Are the Experts?
Source: PLoS One. 2016 Aug 25;11(8):e0161407. doi: 10.1371/journal.pone.0161407 (PMC4999179; doi:10.1371/journal.pone.0161407)
Supplement: S2 Appendix — This is the strategy that was employed on the OVID platform and modified for other databases. (DOCX) [file pone.0161407.s002.docx]

**S1 Table: Search Strategy**

| **Palliative Population** | 1. exp Palliative Care/ | |
| --- | --- | --- |
|  | 1. palliative care.tw | |
|  | 1. exp Terminal Care/ | |
|  | 1. (terminal* adj2 care).tw | |
|  | 1. hospices/ | |
|  | 1. hospice care.tw | |
|  | 1. end stage.tw | |
|  | 1. late stage.tw | |
|  | 1. (advanced adj2 disease*).tw | |
|  | 1. (advanced adj2 illness*).tw | |
|  | 1. terminal* ill*.tw | |
|  | 1. end of life.tw | |
|  | 1. exp Advanced directives/ | |
|  | 1. advanced directive*.tw | |
|  |  | 1. OR 1-14 |
| **Clinicians prediction** | 1. clinica* estimat*.tw. | |
|  | 1. clinica* predict*.tw. | |
|  | 1. physician* estimat*.tw. | |
|  | 1. physician* predict*.tw. | |
|  | 1. nurse* estimat*.tw. | |
|  | 1. nurse* predict*.tw. | |
|  | 1. health* professional* estimat*.tw. | |
|  | 1. health* professional* predict*.tw. | |
|  | 1. doctor* estimat*.tw. | |
|  | 1. doctor* predict*.tw. | |
|  | 1. (estim* adj2 surviv*).tw. | |
|  | 1. exp life expectancy/ | |
|  |  | 1. OR 16-27 |
|  |  | 1. 15 AND 28 |
| **Prognosis** | 1. exp incidence/ | |
|  | 1. exp mortality/ | |
|  | 1. exp follow-up studies/ | |
|  | 1. mortality/ | |
|  | 1. prognos*.tw. | |
|  | 1. predict*.tw. | |
|  | 1. course.tw. | |
|  | 1. exp survival analysis/ | |
|  | 1. survival analysis.tw. | |
|  |  | 1. OR 30-38 |
|  |  | 1. 29 AND 39 |
