# Supplementary material for: A Systematic Review of Predictions of Survival in Palliative Care: How Accurate Are Clinicians and Who Are the Experts?
Source: PLoS One. 2016 Aug 25;11(8):e0161407. doi: 10.1371/journal.pone.0161407 (PMC4999179; doi:10.1371/journal.pone.0161407)
Supplement: S4 Appendix — (DOCX) [file pone.0161407.s004.docx]

| Low risk of bias |
| --- |
| Moderate risk of bias |
| High risk of bias |
